# Virtual Reality for Pain and Anxiety Management in Cardiac Surgery and Interventional Cardiology

**DOI:** 10.1016/j.jacadv.2023.100814

**Published:** 2024-01-05

**Authors:** Sulayman el Mathari, Anne Hoekman, Rohit K. Kharbanda, Amir H. Sadeghi, Rob de Lind van Wijngaarden, Marco Götte, Robert J.M. Klautz, Jolanda Kluin

**Affiliations:** a Department of Cardiothoracic Surgery, Amsterdam University Medical Center, Amsterdam, the Netherlands; b Department of Cardiothoracic Surgery, Leiden University Medical Center, Leiden, the Netherlands; c Department of Cardiothoracic Surgery, Erasmus University Medical Center, Rotterdam, the Netherlands; d Department of Cardiology, Amsterdam University Medical Center, Amsterdam, the Netherlands

**Keywords:** anxiety, cardiac surgery, interventional cardiology, pain, virtual reality

## Abstract

Pain and anxiety are common in patients undergoing cardiac surgery and percutaneous cardiac interventions. Virtual reality (VR) is an emerging non-pharmacological tool for pain and anxiety management. However, its application around cardiac procedures remains relatively unexplored. In this review, we perform a targeted non-systematic literature review to assess the current state-of-the-art of VR for pain and anxiety management in patients undergoing cardiac procedures. Contexts of interest were preprocedural, periprocedural, and postprocedural applications. Existing trials show inconsistent results. The majority of studies in the preprocedural (7 studies, n = 302), periprocedural (1 study, n = 99), and postprocedural stage (4 studies, n = 214) demonstrate significant reduction of pain and anxiety through VR distraction therapy or VR patient education. However, larger-scale trials (2 preprocedural studies [n = 233], 1 periprocedural study [n = 32], 2 postprocedural studies [n = 300]) report no effect. Current literature on effectiveness of VR for pain and anxiety management in cardiac surgery and interventional cardiology remains inconclusive.

## Highlights

• Virtual reality is an emerging non-pharmacological tool for pain and anxiety management.
• Literature on the potential of virtual reality in cardiovascular medicine is scarce.
• Efficacy of virtual reality for pain and anxiety in cardiac interventions remains inconclusive.
• Future research requires larger sample sizes and standardized outcome measures.

Pain and anxiety are prevalent among patients undergoing cardiac surgery and percutaneous cardiac interventions.^1^ Anxiety affects a substantial percentage of cases, ranging from 40% to 80%, across all treatment stages, including preprocedural, periprocedural, and postprocedural phases.^2^, ^3^, ^4^ Pain, on the other hand, is most commonly experienced during the periprocedural and postprocedural periods, affecting approximately 40% to 75% of patients.^5^, ^6^, ^7^ Both pain and anxiety induce the release of stress hormones, such as glucocorticoids, and lead to an increase in pro-inflammatory cytokine levels.^8^, ^9^, ^10^ These physiological changes have been associated with various adverse events, including kidney injury, cerebrovascular events, arrhythmias, myocardial injury, impaired wound healing, delirium, and depression.^11^, ^12^, ^13^

Traditional approaches in pain and anxiety management involve patient education and medical therapy with acetaminophen, nonsteroidal anti-inflammatory drugs and opioids. However, in some cases, these strategies have limited effects and are associated with an elevated risk of side effects, drug tolerance, and dependence.^14^ Adverse reactions associated with these medications include respiratory depression, renal dysfunction, gastrointestinal bleeding, sedation, nausea, vomiting, and constipation.^15^^,^^16^ Consequently, there is a growing need for non-pharmacological interventions to address pain and anxiety in order to mitigate the shortcomings and risks associated with this traditional approach.

**Figure 1:**
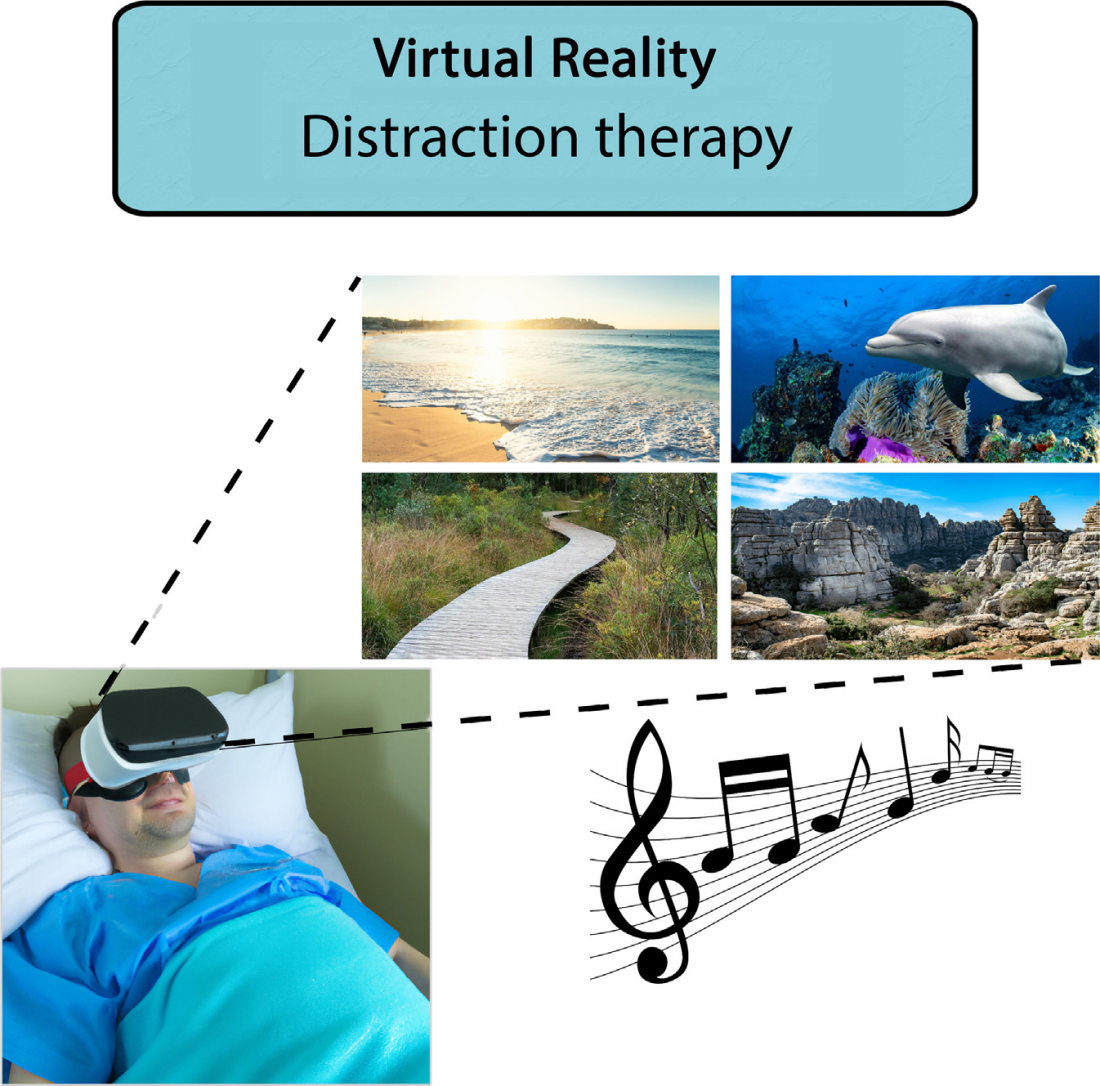
**Virtual Reality Distraction Therapy Is Accomplished by Guiding Patients' Attention Away From the Nociceptive and/or Stressful Stimuli Present in the Environment** It achieves this by immersing them in serene and calming virtual environments accompanied by soothing sounds.

**Figure 2:**
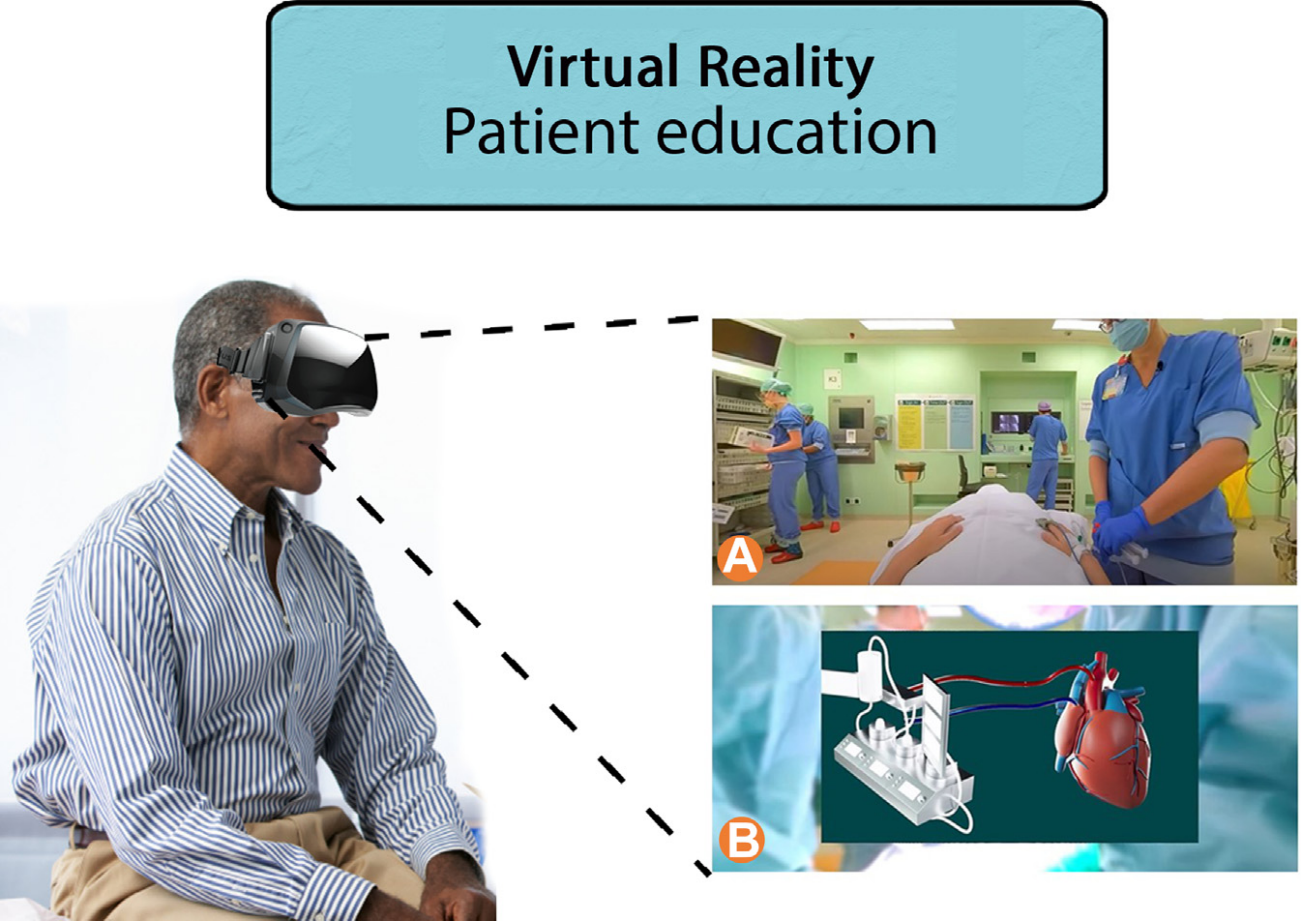
Virtual Reality Education Therapy Involves Creating a Virtual Representation of the Entire Care Process That Patients Will Undergo This can include both a realistic simulation of the practice (A) and a virtual animation depicting the upcoming treatment (B).

Virtual reality (VR) is a modern emerging technique that offers the ability to provide an immersive digital simulation experience. This technology can be effectively utilized for 2 main purposes when it comes to pain and anxiety management: distraction therapy (Figure 1) and patient education (Figure 2). Distraction therapy is accomplished in the form of immersive 3-dimensional (3D) videos, calming music, and breathing exercises.^17^^,^^18^ By incorporating immersive visual and auditory stimuli, VR creates a captivating and serene environment that effectively diverts patients' attention away from the anxiety-inducing aspects of the procedure. This technique can be applied in preprocedural, periprocedural, and postprocedural phase of care. For patient education purposes, VR allows patients to embark on immersive 360° virtual tours that provide a comprehensive representation of their entire awaiting care process or a visual representation of their concerning disease and treatment.^19^ Since this technique aims on education about the awaiting procedure, this is only applied in the preprocedural stage. Various studies have already explored the use of these applications as tools for managing pain and anxiety in patients undergoing cardiac interventions and have reported potential benefits in this regard.^20^, ^21^, ^22^

The objective of this review is to comprehensively examine and elucidate the current and potential impact of VR technology in specifically reducing pain and anxiety across the preprocedural, periprocedural, and postprocedural phases of cardiac surgery and percutaneous cardiac interventions. By thoroughly evaluating and summarizing the existing literature on the application of VR distraction therapy for pain and anxiety management in the cardiovascular field, we aim to add a detailed novel overview to the literature on the concrete potential benefits and limitations associated with the implementation of VR distraction therapy for this specific purpose.

**Figure 3:**
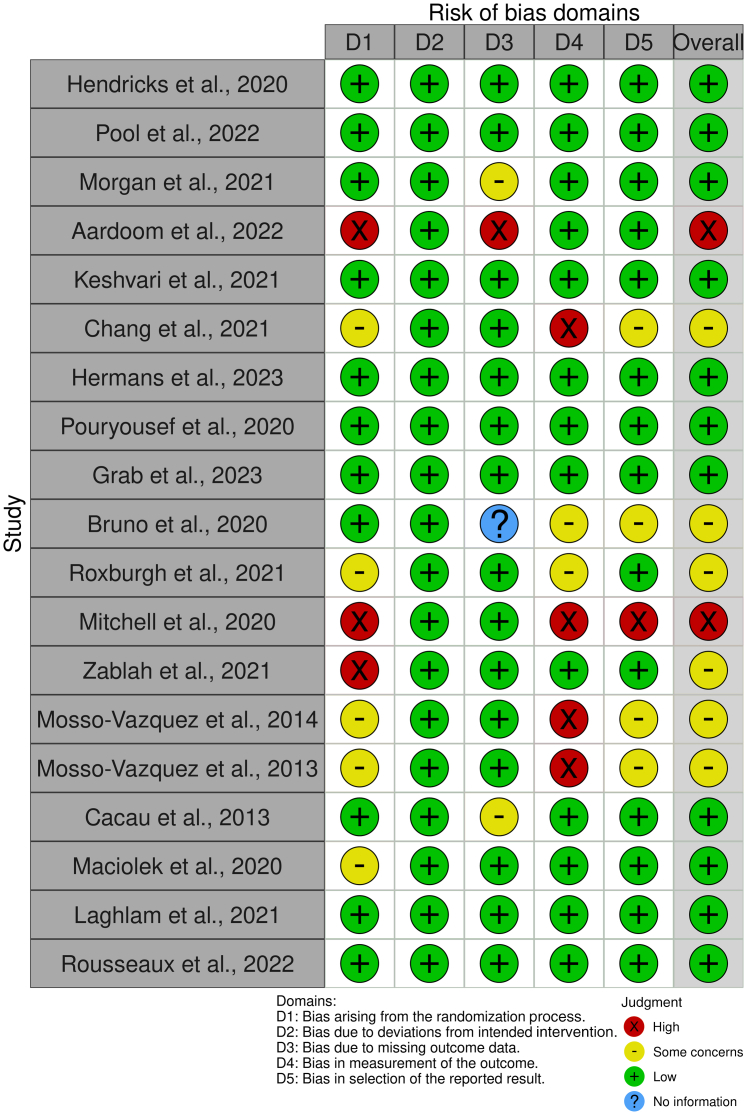
**Risk of Bias Assessment of All Included Studies Using the Cochrane Risk ofBias Tool**

In this review, we performed a targeted non-systematic literature review within the electronic Medline (PubMed) and Google Scholar databases to identify the current state-of-the-art of VR for pain and anxiety management in cardiac surgery and percutaneous cardiac interventions. Contexts of interest were preprocedural, periprocedural, and postprocedural applications. Key search words included: VR, cardiac surgery, percutaneous cardiac interventions, preprocedural, periprocedural and postprocedural, pain management and anxiety management. Additionally, reference lists of all included articles were searched for additional relevant articles. Articles were included if deemed relevant and/or had content related to the aforementioned key words. After the final selection of included articles, a risk of bias assessment (Figure 3) was performed using the Cochrane Risk of Bias Tool.^23^ Below, and in Table 1, we present a comprehensive overview of the articles that were included, focusing on the implementation of VR distraction or education therapy for reducing pain and anxiety in the preprocedural, periprocedural, and postprocedural contexts.

**Table 1:** Overview of VR Studies for Pain and Anxiety Reduction in Cardiac Surgery and Interventional Cardiology by Means of VR Distraction Therapy and Patient Education

Clinical Phase	First Author, Year	Cardiovascular Domain	Study Type	Sample Size (n)	Mean Age (y)	VR Technique	Conclusion of Results
Preprocedural	Hendricks et al, 2020^24^	Cardiac surgery	RCT	20	66.5 ± 8.0	Distraction therapy	Reduced stress (*P* = 0.001) and anxiety (*P* = 0.048) in VR group compared to control group
	Pool et al, 2022^25^	Interventional cardiology	RCT	60	43.8 ± 11.0	Patient education	Lower anxiety (*P* = 0.02) in VR group compared to control group
	Morgan et al, 2021^26^	Interventional cardiology	RCT	64	68.7	Patient education	Lower anxiety (*P* = 0.03) in VR group compared to control group
	Aardoom et al, 2022^19^	Interventional cardiology	Feasibility	8	67.0 ± 7.5	Patient education	Less anxiety in all subjects after VR session (subjective reporting)
	Keshvari et al, 2021^27^	Interventional cardiology	RCT	60	51.6 ± 4.1	Distraction therapy	Lower anxiety (*P* ≤ 0.01) in VR group compared to control group
	Chang et al, 2021^28^	Interventional cardiology	RCT trial	33	58.2 ± 8.5	Patient education	Lower anxiety (*P* ≤ 0.05) in VR group compared to control group
	Hermans et al, 2023^29^	Interventional cardiology	RCT	134	66.3	Patient education	No significant effect of VR on anxiety levels (*P* = 0.4)
	Pouryousef et al, 2020^30^	Interventional cardiology	RCT	90	50.7 ± 8.1	Distraction therapy	Lower anxiety (*P* = 0.001) in VR group compared to control groups
	Grab et al, 2023^31^	Cardiac surgery	RCT	99	64.8 ± 10.9	Patient education	No significant effect of VR on anxiety levels (*P* = 0.76)
Periprocedural	Bruno et al, 2020^32^	Interventional cardiology	RCT	32	83.0 ± 4.8	Distraction therapy	Lower anxiety (*P* = 0.04) in VR group but no difference in pain (*P* = 0.61) compared to control group
	Roxburgh et al, 2021^33^	Interventional cardiology	RCT	99	63.8 ± 10.7	Distraction therapy	Lower pain (*P* = 0.004) in VR group compared to control group
	Mitchell et al, 2020^34^	Interventional cardiology	Case report	1	60	Distraction therapy	The patient reported minimal pain during procedure with VR
	Zablah et al, 2021^35^	Interventional cardiology	Case series	3	14.7 ± 0.5	Distraction therapy	All subjects reported feeling comfortable and minimal pain with VR
Postprocedural	Mosso-Vazquez et al, 2014^36^	Cardiac surgery	Observational	67	Unknown	Distraction therapy	Decrease of pain levels in 88% of all participants after VR session
	Mosso-Vazquez et al, 2013^37^	Cardiac surgery	Observational	22	56.9 ± 10.0	Distraction therapy	Decrease of pain levels in 95% of all participants after VR session
	Cacau et al, 2013^38^	Cardiac surgery	RCT	60	50.6 ± 2.5	Distraction therapy	Lower pain (*P* ≤ 0.05) in VR group compared to control group after 3 d
	Maciolek et al, 2020^39^	Interventional cardiology	RCT	65	59.8 ± 11.8	Distraction therapy	Lower anxiety (*P* ≤ 0.05) in VR group compared to control group
	Laghlam et al, 2021^40^	Cardiac surgery	RCT	200	68.0 ± 7.4	Distraction therapy	No significant differences for pain between VR and control group.
	Rousseaux et al, 2022^41^	Cardiac surgery	RCT	100	66.0 ± 11.5	Distraction therapy	No significant differences for pain and anxiety between VR and control group.

RCT = randomized controlled trial; VR = virtual reality.

## Preprocedural

### Cardiac surgery

Literature on preprocedural application of VR for pain and anxiety management in cardiac surgery is relatively limited compared to the extensive body of research available in the context of interventional cardiology. Currently, the available studies in the field of cardiac surgery are limited to 2 randomized controlled trials (RCTs). The first one investigated the impact of VR distraction therapy on preprocedural anxiety in a cohort of 20 patients undergoing coronary artery bypass grafting procedures.^24^ The study involved random allocation of subjects into either a control group or an intervention group. The intervention group received VR distraction therapy at the preoperative ward on the day of surgery. Outcomes were assessed using the State-Trait Anxiety Inventory (STAI) questionnaire,^42^ which demonstrated notable improvements in the intervention group. Specifically, the intervention group showed significant enhancements in feeling calm (*P* = 0.048) and experienced significant reductions in stress (*P* = 0.001).

The second RCT in this domain assessed the impact of preprocedural VR patient education on anxiety.^31^ This study included 99 subjects undergoing cardiac surgery, who were randomly distributed in 3 groups: 1) control group (n = 34); 2) VR patient education (n = 31); and 3) 3D-printed model education (n = 34). The interventions involved representations of the specific concerning disease and the awaiting treatment. Primary outcomes were assessed using the STAI and visual analog scale (VAS) score both before and after the intervention in each group. This study revealed that only the VR patient education group showed a significant decrease in anxiety levels based on the VAS score (VAS 5.00-4.32, Δ-0.68, *P* < 0.001). However, there were no significant differences in anxiety levels measured by the STAI scores after the interventions between the 3 groups (control: 21.30 ± 5.32, VR: 20.39 ± 5.93, 3D-model: 20.82. ± 5.18, *P* = 0.7638).

### Interventional cardiology

In the domain of interventional cardiology, a recent RCT investigated the effect of VR patient education on preprocedural anxiety in 60 patients planned for percutaneous closure of a patent foramen ovale or atrial septal defect.^25^ Participants were assigned to either the control group, which received conventional preprocedural education, or the intervention group which received VR patient education by means of an immersive virtual tour through their whole awaiting care process. After receiving their assigned education pathway 1 month before their treatment, patients were asked to complete the STAI questionnaire as a measure of the outcome. The results indicated higher levels of anxiety in the control group compared to the intervention group (STAI score 45 ± 11 vs 38 ± 7, *P* = 0.02). A week prior to their procedure, the STAI questionnaire was administered again, revealing increased anxiety levels in the control group, while anxiety levels in the intervention group remained unchanged. These findings not only highlight the immediate impact of the different patient education options on preoperative anxiety but also underscore the distinction in long-term effects between the 2 groups.

Another study assessed the same effect on patients undergoing cardiac catheterization.^26^ Sixty-four patients were randomly assigned to either receive conventional preprocedural care or additional VR patient education. Anxiety levels, procedural knowledge, and satisfaction were evaluated using custom-made questionnaires. The results revealed that patients in the intervention group experienced a significantly greater reduction in anxiety levels from baseline to postprocedure compared to the control group (Δ-5.1 vs Δ-4.0, *P* = 0.03). Additionally, the intervention group demonstrated higher overall satisfaction scores (9.35 vs 8.97, *P* = 0.04) and a better understanding of the procedure (3.88 vs 3.23, *P* < 0.01). A smaller feasibility study in the same population confirmed these results.^19^ A group of 8 patients underwent a virtual tour, providing them with an immersive view of their upcoming cardiac catheterization. Outcomes were assessed using the Presence questionnaire (mean 129.1 ± 13.4), System Usability Scale score (mean 89.1 ± 12), and Client Satisfaction questionnaire (mean 27.1 ± 3.2). All participants reported feeling notably less anxious about the impending procedure after experiencing the immersive VR education program. The same results were reported in a study with 33 patients undergoing atrial fibrillation (AF) ablation.^28^ Twenty-two patients were allocated to the control group and 11 patients to the VR patient education group. Here again, anxiety was measured by means of custom-made questionnaires. Results reported significant lower preprocedural anxiety scores in the VR patient education group compared to the control group (5.0 vs 7.1, *P* < 0.05).

Surprisingly, a relatively large RCT that was published recently did not find a significant effect of VR patient education when compared to conventional education.^29^ This study included 134 patients undergoing AF ablation who were equally distributed among a control and VR patient education group. The assessed primary outcome measure was anxiety, by use of the Amsterdam Preoperative Anxiety and Information Scale (APAIS).^43^ This outcome measure did not differ between the 2 groups (10 [SD 8-13] vs 10 [SD 7-12], *P* = 0.402). Subjects additionally filled in a questionnaire about worries on the procedure, which demonstrated that the intervention group had a significantly lower percentage of patients worrying about the ablation procedure compared to the control group (19.1% vs 40.9%, *P* = 0.006). Nonetheless, it is important to note that this was a nonvalidated questionnaire.

VR distraction therapy, on its turn, showed more consistent beneficial effects. An RCT with 60 patients undergoing coronary angiography studied the effect of preprocedural VR distraction therapy in reducing anxiety.^27^ Prior to undergoing coronary angiography, the intervention group was exposed to an immersive 360-degree VR video featuring immersive natural locations. Conversely, the control group did not partake in this VR session. Anxiety was assessed utilizing the STAI questionnaire. The outcomes indicated a significant reduction in anxiety levels in the intervention group after receiving VR distraction therapy compared to the control group (13.1 vs 15.1, *P* < 0.01). Another RCT involving 90 subjects undergoing cardiac catheterization demonstrated similar results. Here, subjects were divided equally over 3 groups: 1) VR distraction therapy; 2) breathing exercises; and 3) a control group.^30^ Subjects underwent intervention 1 hour before their procedure or received conventional care. The primary outcome was anxiety assessed by the STAI questionnaire. Results showed significantly less anxiety in the VR distraction therapy group compared to the other 2 groups (*P* = 0.001).

## Periprocedural

### Cardiac surgery

VR has demonstrated effectiveness in reducing pain and anxiety when used alongside conscious sedation or local/regional anesthesia during invasive procedures.^44^ However, in case of cardiac surgery, patients are typically placed under general anesthesia, which renders them unconscious and unresponsive to external stimuli, including VR. Consequently, the periprocedural use of VR applications in these patients is not feasible.

### Interventional cardiology

Only 2 studies have reported about the periprocedural application of VR in interventional cardiology. The first study examined the impact of VR distraction therapy on pain and anxiety during transcatheter aortic valve replacement (TAVR) procedures.^32^ The study involved a sample size of 32 participants. The intervention group (n = 16) underwent VR distraction therapy during the TAVR procedure utilizing a head-mounted device, while the control group (n = 16) received conventional treatment. All patients underwent conscious sedation during the intervention. Primary outcomes were periprocedural anxiety and pain, both assessed with the VAS score. The intervention group demonstrated significantly lower levels of periprocedural anxiety (2 [IQR: 0-3.75] vs 5 [IQR: 2-8]; *P* = 0.04). However, there was no significant difference in periprocedural pain levels between both groups (4 [IQR: 3-4.8] vs 4 [IQR: 2-6], *P* = 0.61). A similar RCT was conducted involving 99 subjects undergoing AF ablation.^33^ Forty-eight patients received VR distraction therapy during the procedure, while 51 were treated conventionally. Periprocedural pain was assessed as the primary outcome using the VAS score. Results showed significantly lower periprocedural pain experience in patients who received VR distraction therapy compared to those who were treated conventionally (3.5 ± 1.5 vs 4.3 ± 1.6; *P* = 0.004).

Additionally, a case report and a case series (n = 3) documented the periprocedural utilization of VR distraction therapy for pain and anxiety management during, respectively, emergency pericardiocentesis and diagnostic cardiac catheterization.^34^^,^^35^ In both contexts, the patients received VR distraction therapy throughout the procedures. Subsequently, all patients reported feeling comfortable and experiencing minimal pain during the procedure, attributing it to the distraction provided by the VR experience.

## Postprocedural

### Cardiac surgery

Several studies have examined the impact of postprocedural VR distraction therapy in cardiac surgery patients. An observational study involving 67 subjects described the use of VR distraction therapy to reduce pain after cardiac surgery on the intensive care unit (ICU).^36^ All patients received a 30-minute session of VR distraction therapy on the first day following surgery in the ICU. The primary outcome measure was pain, which was assessed using a custom-made Likert scale. Results showed that 88% of the patients reported a decrease in pain levels (mean Δ-3.75) after the VR intervention. Another study by the same research group supported these results in a smaller group of patients (n = 22).^37^ Patients received the same aforementioned VR intervention during their ICU stay following cardiac surgery and pain was assessed using a custom-made Likert scale survey. Among 22 patients, 21 reported experiencing a decrease in pain levels after undergoing the 30-minute VR intervention. An RCT conducted in the same setting with 100 subjects, yielded contrasting results.^41^ This study assessed the impact of postprocedural VR distraction therapy on pain and anxiety in the ICU. The study design consisted of a control group and 3 intervention arms, which received different treatments: 1) VR distraction therapy; 2) VR distraction therapy with hypnotic audio; and 3) hypnosis by audio. All groups underwent a 20-minute intervention session on the day before and the day after cardiac surgery. Pain and anxiety were evaluated as primary outcomes using a VAS scale. Results showed no significant differences in postprocedural pain and anxiety between all groups. Another RCT consisting of 200 subjects also showed no significant effect of VR distraction therapy on postprocedural pain after cardiac surgery in the ICU.^40^ In this study, subjects were allocated to either a VR distraction therapy group (n = 99) or a control group (n = 101). The primary assessed outcome was postprocedural pain by use of the analgesia/nociception index^45^ and the numeric rate scale (NRS). Results demonstrated no significant differences between the 2 groups in terms of both the analgesia/nociception index score and the NRS score.

Lastly, another RCT was performed in the clinical ward with 60 subjects, evaluating the use of VR distraction therapy for clinical rehabilitation, including pain, after cardiac surgery.^38^ Subjects were equally divided between a control group and intervention group. The VR intervention consisted of immersive exercises such as distractive breathing techniques and physical activity. Primary outcomes were measured using the Nottingham Health Profile questionnaire,^46^ assessing patients’ incapacity across various domains, including pain levels. Results showed no significant difference in pain scores between the groups in the first 2 days. However, after 3 days, there was a significant decrease in pain score among patients in the VR group compared to the controls (*P* < 0.05). Subsequently, patients who received the VR intervention had also a shorter hospital stay compared to those in the control group (9.4 ± 0.5 days vs 12.2 ± 0.9 days, *P* < 0.05).

### Interventional cardiology

In the field of interventional cardiology, there is a single study reporting the effect of postprocedural VR distraction therapy. This study assessed the impact of VR distraction therapy on postprocedural anxiety in patients after percutaneous cardiac angioplasty.^39^ Sixty-five patients were included and divided among a control (n = 33) and intervention group (n = 32). The intervention group received 6 sessions of VR distraction therapy during the first 3 days of the postprocedural stage. Each VR session lasted for 20 minutes. The primary outcomes for anxiety were measured using the STAI questionnaire. Results showed that both groups experienced a reduction in anxiety after 3 days. However, this reduction was significantly greater in the intervention group compared to the control group (STAI score reduction −4.89 vs −2.72, *P* ≤ 0.05).

## Discussion

Cardiac procedures, encompassing both cardiac surgery and percutaneous cardiac procedures, often result in the experience of pain and/or anxiety.^1^, ^2^, ^3^, ^4^ These 2 side effects are interconnected, creating a detrimental cycle when left untreated.^47^ Consequently, these factors may have a negative impact on clinical outcomes. Hence, it becomes imperative to provide optimal management for pain and anxiety in this specific patient population. This paper explores the potential of VR as a nonpharmacological tool to reduce pain and anxiety among cardiac patients before, during, and after surgical and percutaneous cardiovascular interventions (Central Illustration).

**Central Illustration:**
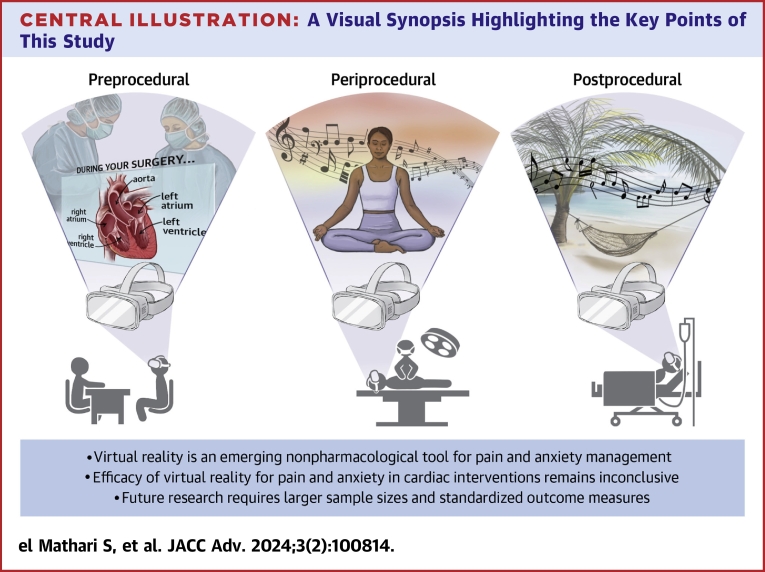
Virtual Reality for Pain and Anxiety Management in Cardiac Surgery and Interventional Cardiology

Trials evaluating the use of VR in preprocedural settings for cardiac surgery and interventional cardiology show inconsistent results regarding the effects on pain and anxiety. While the majority of trials (7 studies with a total number of 302 subjects) have demonstrated promising potential in significantly reducing preprocedural pain and anxiety through VR distraction therapy or VR patient education,^19^^,^^24^, ^25^, ^26^, ^27^, ^28^^,^^30^ 2 larger-scale trials (N = 233) have reported no significant effect^29^^,^^31^ (Table 1).

This same inconsistency persists in the periprocedural assessment of VR distraction therapy for pain and anxiety during TAVR and AF ablation procedures. One RCT (n = 32) reported no significant effect on pain,^32^ while another RCT (n = 99) demonstrated a significant reduction of periprocedural pain.^33^ The latter is further supported by the reported case series showing beneficial effects of periprocedural VR distraction therapy in individual patients during cardiac catheterization (n = 3) and pericardiocentesis (n = 1).^34^^,^^35^

The postprocedural stage presents a significant opportunity to apply VR distraction therapy for pain and anxiety reduction. However, in this context as well, the existing literature presents conflicting results. While the majority of trials (4 studies with a total number of 214 subjects) report a significant reduction in postprocedural pain and anxiety with VR distraction therapy,^36^, ^37^, ^38^, ^39^ 2 larger-scale trials (shared total of 300 subjects) show no significant difference compared to conventional treatment.^40^^,^^41^

It is important to acknowledge the variability in these reported findings throughout all stages of cardiac care. The conflicting results observed in trials investigating VR for pain and anxiety in cardiac surgery and interventional cardiology can be attributed to several factors. Firstly, the effectiveness of VR interventions may vary depending on factors such as the duration and number of the intervention(s), technical quality of the intervention, patient characteristics such as age, and the specific clinical context in which the tool is utilized. For instance, a wide variety of technical tools is employed in VR distraction therapy, yielding possibly a corresponding spectrum of outcomes and effects. Secondly, it is important to recognize that the majority of studies included in this paper have small sample sizes, which raises concerns about the reliability of the reported outcomes. Thirdly, there is inconsistency in the way outcomes are measured and reported. Many studies utilize nonvalidated custom-made scores, further casting doubt on the reliability of the findings. Furthermore, the performed risk of bias assessment reveals notable concerns in at least 8 out of the 19 studies incorporated, emphasizing the imperative need for additional qualitative research.

Nevertheless, the efficacy of VR for pain and anxiety management in cardiac surgery and interventional cardiology remains inconclusive. To address this, future research should prioritize conducting trials with larger sample sizes to enhance statistical power and improve the generalizability of findings. Additionally, studies should focus on using validated instruments (STAI, APAIS, NRS) to measure outcomes, ensuring more reliable and standardized assessments. Furthermore, it is important to note that current research primarily relies on subjective pain experience assessments through questionnaires, which may raise concerns about the validity of these outcomes. Pain and anxiety are a complex phenomenon influenced by biological, social, and psychological factors, and their manifestation can vary among individuals. Therefore, incorporating objective measures such as heart rate variability and changes in blood pressure would provide a more comprehensive evaluation of the impact of VR on pain and anxiety. By structurally considering these recommendations, future studies can provide a more robust understanding of the effectiveness of VR in managing pain and anxiety in cardiac procedures.

Moreover, the cost-effectiveness of utilizing VR for pain and anxiety management must be carefully assessed. This evaluation encompasses not only the expenses associated with software and hardware but also the time commitment required for integrating VR devices into daily clinical practices. Given that nurses are already engaged in essential clinical duties, introducing additional staff for the implementation of VR devices would incur additional costs. While other medical fields have demonstrated economic benefits of VR,^48^^,^^49^ including reduced average costs per quality-adjusted life year through shorter hospitalization, decreased medication usage, and lower risk of clinical readmission, the cardiovascular field has yet to assess these factors. Conducting cost-effectiveness analyses specific to cardiac procedures would provide valuable insights into the economic impact of implementing VR interventions. Such analyses can help health care providers and policymakers make informed decisions about the adoption and integration of VR technology into routine cardiac care, considering both its clinical efficacy and financial implications.

We acknowledge that the narrative nature of this review is a limitation. While a thorough and comprehensive literature review was conducted, it is important to note that it was not a systematic literature review. This is primarily due to the scarcity of available data on this topic, which hinders the feasibility of conducting a comprehensive systematic review with a meta-analysis. Yet, we can conclude that the current literature on the efficacy of VR applications for pain and anxiety management in cardiac surgery and interventional cardiology remains inconclusive. The conflicting results and limitations of the available studies highlight the need for future research with larger sample sizes and the standardized use of validated outcome instruments. It is important to recognize that VR is evolving rapidly, and as it continues to advance, the potential for VR in pain and anxiety management may expand in the future. Further exploration and investigation in this field are warranted to fully understand the benefits and limitations of VR in improving patient outcomes and experiences.

## Funding support and author disclosures

The authors have reported that they have no relationships relevant to the contents of this paper to disclose.

## References

[1] Vingerhoets G. (1998). Perioperative anxiety and depression in open-heart surgery. Psychosomatics.

[2] Delewi R., Vlastra W., Rohling W.J. (2017). Anxiety levels of patients undergoing coronary procedures in the catheterization laboratory. Int J Cardiol.

[3] Ayasrah S.M., Ahmad M.M. (2016). Educational video intervention effects on periprocedural anxiety levels among cardiac catheterization patients: a randomized clinical trial. Res Theory Nurs Pract.

[4] Osama Y., Amer R., Fawzy H., Shama G. (2019). Psychiatric disturbances in patients undergoing open-heart surgery. Middle East Curr Psychiatry.

[5] Miozzo A.P., Stein C., Bozzetto C.B., Plentz R.D.M. (2016). Massage therapy reduces pain and anxiety after cardiac surgery: a systematic review and meta-analysis of randomized clinical trials. Clin Trials Regul Sci Cardiol.

[6] Guimaraes-Pereira L., Reis P., Abelha F., Azevedo L.F., Castro-Lopes J.M. (2017). Persistent postoperative pain after cardiac surgery: a systematic review with meta-analysis regarding incidence and pain intensity. Pain.

[7] Choiniere M., Watt-Watson J., Victor J.C. (2014). Prevalence of and risk factors for persistent postoperative nonanginal pain after cardiac surgery: a 2-year prospective multicentre study. CMAJ.

[8] Baytar A.D., Bollucuo Lu K. (2023). Effect of virtual reality on preoperative anxiety in patients undergoing septorhinoplasty. Braz J Anesthesiol.

[9] Desai R.G., Muntazar M., Goldberg M.E. (2009). Strategies for managing perioperative hypertension. Curr Hypertens Rep.

[10] Varon J., Marik P.E. (2008). Perioperative hypertension management. Vasc Health Risk Manag.

[11] Stengrevics S., Sirois C., Schwartz C.E., Friedman R., Domar A.D. (1996). The prediction of cardiac surgery outcome based upon preoperative psychological factors. Psychol Health.

[12] Munafo M.R., Stevenson J. (2001). Anxiety and surgical recovery. Reinterpreting the literature. J Psychosom Res.

[13] Christian L.M., Graham J.E., Padgett D.A., Glaser R., Kiecolt-Glaser J.K. (2006). Stress and wound healing. Neuroimmunomodulation.

[14] Sloot S., Boland J., Snowden J.A. (2015). Side effects of analgesia may significantly reduce quality of life in symptomatic multiple myeloma: a cross-sectional prevalence study. Support Care Cancer.

[15] Benyamin R., Trescot A.M., Datta S. (2008). Opioid complications and side effects. Pain Physician.

[16] Rainsford K.D. (1999). Profile and mechanisms of gastrointestinal and other side effects of nonsteroidal anti-inflammatory drugs (NSAIDs). Am J Med.

[17] Olbrecht V.A., O'Conor K.T., Williams S.E. (2021). Guided relaxation-based virtual reality for acute postoperative pain and anxiety in a pediatric population: pilot observational study. J Med Internet Res.

[18] Patterson D.R., Wiechman S.A., Jensen M., Sharar S.R. (2006). Hypnosis delivered through immersive virtual reality for burn pain: a clinical case series. Int J Clin Exp Hypn.

[19] Aardoom J.J., Hilt A.D., Woudenberg T., Chavannes N.H., Atsma D.E. (2022). A preoperative virtual reality app for patients scheduled for cardiac catheterization: pre-post questionnaire study examining feasibility, usability, and acceptability. JMIR Cardio.

[20] Stewart D., Mete M., Groninger H. (2019). Virtual reality for pain management in patients with heart failure: study rationale and design. Contemp Clin Trials Commun.

[21] Sadeghi A.H., Mathari S.E., Abjigitova D. (2022). Current and future applications of virtual, augmented, and mixed reality in cardiothoracic surgery. Ann Thorac Surg.

[22] Jozwik S., Cieslik B., Gajda R., Szczepanska-Gieracha J. (2021). Evaluation of the impact of virtual reality-enhanced cardiac rehabilitation on depressive and anxiety symptoms in patients with coronary artery disease: a randomised controlled trial. J Clin Med.

[23] Higgins J.P.T., Sterne J.A.C., Savovic J. (2016). A revised tool for assessing risk of bias in randomized trials. Cochrane Database Syst Rev.

[24] Hendricks T.M., Gutierrez C.N., Stulak J.M., Dearani J.A., Miller J.D. (2020). The use of virtual reality to reduce preoperative anxiety in first-time sternotomy patients: a randomized controlled pilot trial. Mayo Clin Proc.

[25] Oudkerk Pool M.D., Hooglugt J.Q., Kraaijeveld A.J. (2022). Pre-procedural virtual reality education reduces anxiety in patients undergoing atrial septal closure – results from a randomized trial. Int J Cardiol Congenit Heart Dis.

[26] Morgan H., Nana M., Phillips D., Gallagher S. (2021). The effect of a VIrtual RealiTy immersive experience upon anxiety levels, procedural understanding, and satisfaction in patients undergoing CArdiac CaTHeterization: the VIRTUAL CATH trial. J Invasive Cardiol.

[27] Keshvari M., Yeganeh M.R., Paryad E., Roushan Z.A., Pouralizadeh M. (2021). The effect of virtual reality distraction on reducing patients' anxiety before coronary angiography: a randomized clinical trial study. Egypt Heart J.

[28] Chang S.L., Kuo M.J., Lin Y.J. (2021). Virtual reality-based preprocedural education increases preparedness and satisfaction of patients about the catheter ablation of atrial fibrillation. J Chin Med Assoc.

[29] Hermans A.N.L., Betz K., Verhaert D.V.M. (2023). 360 degrees virtual reality to improve patient education and reduce anxiety towards atrial fibrillation ablation. Europace.

[30] Pouryousef F., Navidian A., Ghahdarijani O.R., Yaghoubinia F. (2021). Comparing the effect of virtual reality and rhythmic breathing on the anxiety of the patients undergoing coronary angiography. Intern Med Today.

[31] Grab M., Hundertmark F., Thierfelder N. (2023). New perspectives in patient education for cardiac surgery using 3D-printing and virtual reality. Front Cardiovasc Med.

[32] Bruno R.R., Lin Y., Wolff G. (2020). Virtual reality-assisted conscious sedation during transcatheter aortic valve implantation: a randomised pilot study. EuroIntervention.

[33] Roxburgh T., Li A., Guenancia C. (2021). Virtual reality for sedation during atrial fibrillation ablation in clinical practice: observational study. J Med Internet Res.

[34] Mitchell A.R.J., Gibbs A., Birge M., Richardson E. (2020). Virtual reality as a therapy adjunct during emergency pericardiocentesis. Eur Heart J Case Rep.

[35] Zablah J.E., Rodriguez S.A., Leahy R., Morgan G.J. (2022). Implementation of virtual reality for patient distraction during diagnostic cardiac catheterisation. Cardiol Young.

[36] Mosso-Vazquez J.L., Gao K., Wiederhold B.K., Wiederhold M.D. (2014). Virtual reality for pain management in cardiac surgery. Cyberpsychol Behav Soc Netw.

[37] Mosso Vázquez J.L., Santander A., Mosso J.L., Gao K., Wiederhold B.K., Wiederhold M.D. (2013). Using cybertherapy to reduce postoperative anxiety in cardiac recovery intensive care units. J Anesth Clin Res Theory Nurs Pract.

[38] Cacau L.D.P., Oliveira G.U., Maynard L.G. (2013). The use of the virtual reality as intervention tool in the postoperative of cardiac surgery. Rev Bras Cir Cardiovasc.

[39] Maciolek J., Wasek W., Kaminski B., Piotrowicz K., Krzesinski P. (2020). The impact of mobile virtual reality-enhanced relaxation training on anxiety levels in patients undergoing cardiac rehabilitation. Kardiol Pol.

[40] Laghlam D., Naudin C., Coroyer L. (2021). Virtual reality vs. Kalinox(R) for management of pain in intensive care unit after cardiac surgery: a randomized study. Ann Intensive Care.

[41] Rousseaux F., Dardenne N., Massion P.B. (2022). Virtual reality and hypnosis for anxiety and pain management in intensive care units: a prospective randomised trial among cardiac surgery patients. Eur J Anaesthesiol.

[42] Zsido A.N., Teleki S.A., Csokasi K., Rozsa S., Bandi S.A. (2020). Development of the short version of the spielberger state-trait anxiety inventory. Psychiatry Res.

[43] Celik F., Edipoglu I.S. (2018). Evaluation of preoperative anxiety and fear of anesthesia using APAIS score. Eur J Med Res.

[44] Faruki A.A., Nguyen T.B., Gasangwa D.V. (2022). Virtual reality immersion compared to monitored anesthesia care for hand surgery: a randomized controlled trial. PLoS One.

[45] Boselli E., Jeanne M. (2014). Analgesia/nociception index for the assessment of acute postoperative pain. Br J Anaesth.

[46] Falcoz P.E., Chocron S., Mercier M., Puyraveau M., Etievent J.P. (2002). Comparison of the Nottingham Health Profile and the 36-item health survey questionnaires in cardiac surgery. Ann Thorac Surg.

[47] Ploghaus A., Narain C., Beckmann C.F. (2001). Exacerbation of pain by anxiety is associated with activity in a hippocampal network. J Neurosci.

[48] Ashmore J., Di Pietro J., Williams K. (2019). A free virtual reality experience to prepare pediatric patients for magnetic resonance imaging: cross-sectional questionnaire study. JMIR Pediatr Parent.

[49] Pot-Kolder R., Veling W., Geraets C. (2020). Cost-effectiveness of virtual reality cognitive behavioral therapy for psychosis: health-economic evaluation within a randomized controlled trial. J Med Internet Res.

